# How we remember the emotional intensity of past musical experiences

**DOI:** 10.3389/fpsyg.2014.00911

**Published:** 2014-08-15

**Authors:** Thomas Schäfer, Doreen Zimmermann, Peter Sedlmeier

**Affiliations:** Department of Psychology, Chemnitz University of TechnologyChemnitz, Germany

**Keywords:** music, intensity, peak–end, temporal integration, duration neglect

## Abstract

Listening to music usually elicits emotions that can vary considerably in their intensity over the course of listening. Yet, after listening to a piece of music, people are easily able to evaluate the music's overall emotional intensity. There are two different hypotheses about how affective experiences are temporally processed and integrated: (1) all moments' intensities are integrated, resulting in an averaged value; (2) the overall evaluation is built from specific single moments, such as the moments of highest emotional intensity (peaks), the end, or a combination of these. Here we investigated what listeners do when building an overall evaluation of a musical experience. Participants listened to unknown songs and provided moment-to-moment ratings of experienced intensity of emotions. Subsequently, they evaluated the overall emotional intensity of each song. Results indicate that participants' evaluations were predominantly influenced by their average impression but that, in addition, the peaks and end emotional intensities contributed substantially. These results indicate that both types of processes play a role: All moments are integrated into an averaged value but single moments might be assigned a higher value in the calculation of this average.

## Introduction

Music is used in everyday life for numerous purposes, one of the most important of which is the regulation of moods and emotions (e.g., Sloboda et al., 2001; Juslin and Laukka, 2004; Saarikallio and Erkkilä, 2007; Schäfer et al., 2013). Hence, it comes as no surprise that music's potential to regulate moods and emotions has an influence on how pleasant people perceive a piece of music to be (see Sedlmeier and Schäfer, 2013). Practically, this means that people will actively choose to buy and/or listen to pieces of music they know have emotion-regulating or mood-regulating potential. But how do listeners remember the emotional impact of music they heard in the past? Do they remember single extraordinary moments or elements of pieces or is there something like an averaged remembered value? There has not been much empirical research on these questions. In the present article, we present a study on the evaluation of past musical experiences. When listening to a piece of music there is a time course of emotional intensity. On the one hand, emotional intensity can change from moment to moment (reach peaks and troughs) and does not need to be constant over the time course of the musical experience. On the other hand, listeners are also able to evaluate the overall emotional intensity of the whole musical experience. Our study was intended to analyze how listeners take this time course of emotional intensity into account to arrive at a subsequent overall evaluation of emotional intensity. Knowledge of this process should be of interest to performers and composers. It could help them arrange pieces of music that leave an emotionally intense overall memory.

In the following, we first discuss the theoretical background and empirical findings regarding the subjectively felt emotional intensity of affective experiences in general. Afterward, we do the same for studies that used music as the affective stimulus. Finally, we present the results of our own study.

Note that we use the term *musical experience*, which does not include activities such as playing an instrument, singing, or remembering or reading about music, but only the specific act of focused listening. Thus, the term is comparable to what Behne (1997, p. 143) called *Musikerleben*: “the sum of psychic processes which accompany the experience of music in situations when music is in the focus of interest: When a person is not only hearing, but listening to and appreciating music.” The feeling of emotions is one of the most important parameters of musical experiences. The absolute extent of emotions felt during listening to music (regardless of whether they are positive or negative) is defined as *emotional intensity*.

## Evaluation of affective experiences in general

### Theoretical background

Affective experiences can be of a short- or long-term nature. Regarding short-term experiences, the momentary affect can be easily evaluated at the instant it unfolds. On the other hand—and this is what the present study is about—there are *experiences of longer duration and varying affective intensity*. Forming an overall evaluation of these experiences requires generating a *global overall value* of affective intensity (Varey and Kahneman, 1992). The evaluation of momentary affect happens with little reflection. The retrospective evaluation of past affective experiences, however, requires recall and overall assessment that involves integrating all—or only some—moments of that experience (Fredrickson, 2000). There are two mental processes involved in retrospective evaluation: *memory* and *evaluation* of past affective experiences (Kahneman et al., 1993). To understand the evaluation of emotional intensity of past musical experiences it is useful to explore *how people retrospectively remember and evaluate past affective experiences of long duration and varying affective intensity in general*.

There are two competing theories of how the storage of past affective experiences in memory and the process of evaluation occur (Fredrickson and Kahneman, 1993). The first suggestion—sometimes illustrated by the metaphor of film—is that all details of an experience are comprehensively represented in memory. Affective intensity is stored as a function of time, while time itself might or might not be stored in memory, as well. Consequently, retrospective evaluation of overall affective intensity is based on the temporal integration of the affective intensities of a certain number of single “moments.” This is why the model is referred to as the *temporal integration model*. From this point of view, the overall evaluation of experienced affect should strongly depend on the relative duration of specific strengths of affect. That is, the retrospective overall evaluation of an experience is best predicted by the average of all single moments because the longer the affect is relatively strong, the larger the average becomes over time, and the longer the affect is relatively weak, the smaller the average becomes over time^1^.

The second theory—sometimes illustrated by the metaphor of a collection of snapshots—is that the whole experience is represented in memory *fragmentally*. Only intensities of specific moments are stored, and time is not represented in memory at all. Consequently, overall retrospective evaluation of the whole affective experience is based on (the average of) just a few moments' affective intensities. From this point of view, duration of the affective experience should not have any influence on overall evaluation, which has been called *duration neglect*.

What might these moments be that listeners remember more than other moments? There are three specific moments of a musical experience that might be of specific importance: the onset moment, the moment of highest emotional intensity (referred to as the *peak*), and the end of the experience. In addition, any combination of these three moments could play a role in the overall evaluation. Specifically, many scholars have considered the combination of the peak moment and the end moment very important—a conjecture that has become known as the *peak*–*end* rule (see Kahneman, 1999, 2011): When people evaluate a past experience they might pay attention above all to two things, how it felt at the peak and at the end; other information (e.g., net pleasantness or unpleasantness, duration of the experience) is not lost but is simply not taken into account. Fredrickson (2000) has reasoned why people should build their overall evaluation from the peak and the end of an experience: These two moments usually are special carriers of personally relevant meaning. The peak indicates how enjoyable or how threatening an experience can get. The end conveys the information that the experience can be survived. The peak–end rule is considered being used as a simple heuristic, which can be very useful even though it might also lead to mistakes.

### Empirical findings

The questions of how affective experiences are represented in memory and how people evaluate them have been examined in numerous studies. There is a common method used in these studies: Participants' actual moment-to-moment ratings of affective intensity are continuously measured during the experience. Subsequently, the retrospective evaluation of the overall affective intensity is assessed by participants' ratings. Then, the relationship between the recorded time course of the affective experience and the retrospective overall evaluation is analyzed using correlation or regression methods.

A considerable number of studies have supported the idea of the peak–end rule (for an overview, see Kahneman, 2011). In a study on pleasant and aversive film clips, Fredrickson and Kahneman (1993) demonstrated that peak affect and end affect had a remarkable effect on participants' global retrospective ratings of each film's affective intensity. Moreover, the results were not influenced by the specific time delay (which could be shorter or longer) between the end of the actual experience and the subsequent retrospective evaluation, indicating that the global ratings were stable over time. In addition, sessions with and without moment-to-moment ratings resulted in similar overall evaluations. This finding was an important justification for the research method applied, because it demonstrated that moment-to-moment ratings did not distort salience or memories of specific moments. In a study on pain induced by immersing a hand in cold ice water (Kahneman et al., 1993), participants had to endure a short trial (60 s of 14°C) and a long trial (60 s of 14°C + 30 s of 15°C). The long trial was objectively more painful because it included a greater amount of total pain, but it had a better end than the short trial. Surprisingly, participants evaluated the longer trial as less painful, even though they were able to judge the durations correctly. This also led to the conclusion that people put particularly high value on the end of an affective experience. Also, in a study on pain induced by colonoscopy and lithotripsy, Redelmeier and Kahneman (1996) demonstrated the importance of the end of the painful experience. Stone et al. (2000) examined rheumatoid arthritis and found that peak–end was a better predictor for the evaluation of overall pain than the global average of all single moments. Neither peak nor end alone were as powerful predictors as their average. Schreiber and Kahneman (2000) found evidence for the peak–end rule in a study on aversive sounds, as did Langer et al. (2005) in a study on payment sequences and Do et al. (2008) in a study on material gains.

Yet, there are a number of studies that did not confirm the peak–end rule. In a study on pleasant advertisements, Baumgartner et al. (1997) found that peak and end, as separate factors of the experience, were better predictors of the overall evaluation than was their average. When investigating the enjoyment of meals, Rode et al. (2007) did not find either peak or end to be more important than any other element of the time course. Robinson et al. (2011) found that only the peak of the moment-to-moment enjoyment predicted the overall enjoyment of meals. Kemp et al. (2008) studied affective autobiographical events and found that participants did not remember the peaks and troughs of the intensity of happiness during their holidays better than other moments. Cojuharenco and Ryvkin (2008) demonstrated that average and peak–end are comparable in terms of their role in predicting the overall evaluation of experiences and that neither showed an advantage over the other.

In sum, studies with nonmusic stimuli have left an unclear picture. Although most have revealed that the overall evaluation of affective intensity can be well predicted by the average of the most intense moment and the moment at the end of the experience, there are also studies that did not support the validity of this peak–end rule. Notably, almost all of the data supporting the peak–end rule come from negative experiences, so it is questionable if those results can be transferred to musical experiences. Specifically, it is not clear if the consolidation of pain experiences is comparable to the consolidation of musical experiences regarding, for instance, habituation processes or psychological coping mechanisms. It is hard to tell from all the mentioned studies if the remembrance and overall evaluation of a past experience rely on integrated moments, such as the average, or on distinct moments, such as peak or end. We now describe research that incorporated music as a stimulus.

## Evaluation of affective experiences with music

### Theoretical background

Listening to music is characterized by varying moment-to-moment emotional intensity. The characteristics of an affective musical experience and the subsequent retrospective evaluation of its overall emotional intensity are consistent with the characteristics of the above-defined general process of remembering and evaluating past affective experiences of long duration and varying affective intensity. Hence, the theoretical approaches to explaining this general process have been utilized to investigate the special case of evaluating the emotional intensity of past musical experiences.

Typically, the intensity of music-induced affect has been recorded with the use of a dial, slider, or pressure-sensitive button. Data are recorded by having participants manipulate the device according to the intensity of the emotions they are feeling. Madsen (1990; see also Madsen et al., 1993) justified the use of such methods instead of asking people about their subjective experience: Children, handicapped people, and untrained musicians in general often simply do not have an ability for high-level verbal abstraction and find it difficult to express musical changes they hear or feel. Moreover, people might not be able to verbally document their musical experience while actually listening. The act of verbally reporting one's own responses while listening may interfere with the actual experience, and the experience itself may cease or stop quickly when the focus of attention is drawn away from it. Hence, the advantage of moment-to-moment measurement is that the listener is able to track the focus of attention on the music without speaking or writing.

### Empirical findings

The relationship of actual moment-to-moment musical experience and the overall evaluation of those experiences has not been well examined so far. Evidence was provided first by Sloboda and Lehmann (2001), whose study did not focus on *felt* emotions but on emotions participants *perceived* in the music. Nevertheless, they found that the average of all single moments of musical experience correlated with the subsequent global rating by *r* = 0.50. Duke and Colprit (2001) investigated the magnitude of musical moment-to-moment intensity and found that the average of all ratings is different from the overall *post-hoc* rating. However, they did not calculate the covariation of these measures.

Rozin et al. (2004) investigated how remembered overall musical affect is derived from moment-to-moment musical affect. Their participants listened to various music selections of different durations (i.e., each song had a different number of single “moments”). After measuring moment-to-moment affective intensity ratings during each song, the authors measured remembered overall affective intensity of each musical selection. Based on several predictors (average of all single moments' intensities, sum of all single moments' intensities, onset intensity, offset intensity, minimum intensity, peak intensity, and sum of peak and offset intensities), they found that remembered intensity of affect was most highly correlated with peak (*r* = 0.82), peak–end (*r* = 0.81), and average (*r* = 0.80). However, the authors did not run a regression analysis to identify which of the potential parameters accounted for a significant proportion of variance of the overall evaluation. Nonetheless, they concluded that their data did not support the peak–end rule because peak–end was most highly correlated with the overall judgment for only 3 of the 20 participants. Not least, they found a slope effect: large, positive differences in the emotional intensity between consecutive moments were also a reliable predictor of the remembered overall intensity.

The results of Rozin et al. (2004) provide a valuable piece of evidence of how listeners generate an overall evaluation of the emotional intensity of musical experiences. However, there are a number of concerns about potential methodological limitations in the Rozin et al. (2004) study, particularly the choice of stimuli and measurement, which we addressed in the present study. (1) The authors used songs that were known to the participants as well as songs that were unknown. Regardless of whether a song was known or unknown, participants always listened to the music one time to become familiar with the song and a second time for measurement. It is questionable if this is an adequate procedure for achieving comparable familiarity with known and unknown songs. It may be more suitable to use only unknown music to ensure a controlled design and to explore cognitive processes based on “first impressions.” (2) Rozin et al. (2004) provided their participants with a fixed order of songs, which may lead to order effects as a result of participant fatigue and variability of motivation. (3) They used just short extracts of the songs with limited time frames of around 40 s, which may not represent naturalistic listening and may hinder valid generalizations. (4) The authors did not perform a regression analysis or calculate partial correlations but reported only the first-order correlations between the overall judgment and the parameters that potentially influence it, for each participant. Therefore, the specific proportions of variance explained by the most important parameters are not known. Examining the specific impact of potential parameters (while controlling for the impact of others) would allow for determining the relative importance of the potential strategies for extracting an overall emotional evaluation from the time course of moment-to-moment evaluations. (5) The slope effect Rozin et al. (2004) identified in their Discussion suggests that the *variation* of the time course of the moment-to-moment experience might be an additional parameter that influences the overall evaluation and therefore should be incorporated in the calculations. (6) The authors followed a common procedure when calculating peak–end values, that is, building the average of the peak value (the moment of highest intensity) and the end value. However, consistent with the central claim of duration neglect theory, one might expect not a single peak but the average impression of all moments that stand out (*multiple peaks*) as well as the number of these moments (*number of multiple peaks*) to determine the overall evaluation.

## Aim of the present study

By way of summary, the results of studies about the remembrance and evaluation of the emotional intensity of past musical experiences are as inconclusive as those from the nonmusic studies discussed above. From these lines of research, it is still hard to tell if retrospective overall affect is based on an integration of the whole experience, on just specific single moments, or on the average of such moments. In the present study, we attempted to answer this question while circumventing the limitations of past research we have addressed above. We took into account multiple parameters of the temporal profile of emotional intensity of musical experiences: beginning, peak, average of multiple peaks, number of multiple peaks, end, peak–end, multiple peaks–end, sum, average, and variation (see below for the calculation of these parameters). Since music listening is an experience that unfolds over time, we argue that listeners would process an evaluation of the emotional intensity by continuously updating their felt moment-to-moment affect. That is, we predict that listeners average their experiences over time and use the averaged value at the end of the experience as an overall evaluation. There may or may not be moments such as the peak or the end of the experience that are weighted more strongly in the process of averaging. As we cannot draw a more specific hypothesis about this process of weighting based on previous research, we treat this issue as an explorative question. Although it is plausible that the remembrance of only some specific moments that might carry important information about an experience (as do the peak[s] and the end) is a parsimonious heuristic, this is unlikely to be the whole story. Specifically, there is no reason to expect that any information during an experience would be systematically ignored. It is more reasonable to expect that a process of continuous updating occurs over the course of an experience, which would most easily be gathered by the average of the experience of all moments.

## Methods

### Participants

Participants (*N* = 54) were psychology students, 44 (81.5%) female, 10 (18.5%) male. They ranged in age from 18 to 35 years (*M* = 22.3 years, *SD* = 3.2). Nineteen (35%) were involved in some kind of musical activity (singer, choir, band, orchestra, etc.) and 35 (65%) were not. Music was an important part of life for all of them (Min = 4, Max = 9, *M* = 7.52, *SD* = 1.37, on a scale from 1—*not important at all*, to 9—*very important*) and they varied in self-rated musicality (Min = 1, Max = 9, *M* = 5.48, *SD* = 2.23, on a scale from 1—*not musical at all*, to 9—*very musical*). The students received course credits for their participation.

### Ethical approval

The study was performed in accordance with relevant institutional and national guidelines and regulations (Chemnitz University of Technology, 2002; Deutsche Gesellschaft für Psychologie [German Psychological Society], 2005). Informed consent was obtained from all participants. Anonymity of participants and confidentiality of their data were ensured.

### Stimuli

Participants listened to a selection of 11 complete songs of different durations and genres (pop, rock, instrumental rock, hip hop, electro, jazz, classical, emo, reggae, Latino). The songs were thought to be unknown to them. As individual musical experiences are very subjective, can clearly differ, and were not evident before the end of the study, the choice of songs was based on the authors' own and two other raters' subjective judgments. Each song had to fulfill the following criteria: During the song, elicited emotional intensity should not be constant but should reach peaks and troughs in order to maximize the variance within a song and thus between the different parameters of a song (e.g., beginning, peak, end, peak–end, average). Moreover, different songs were chosen to elicit different levels of emotional intensity; that is, some songs were intended to be generally more emotionally intense than others in order to maximize the variance between the songs. Songs, together with their performers and lengths, are listed in Table 1.

**Table 1:** **Songs used in the study**.

**Song**	**Performer**	**Length (min:s)**	**Frequency**
Making Love Out of Nothing at All	Bonnie Tyler	7:49	44
The Post War Dream	Pink Floyd	3:01	49
A Two Hearts Spell	Claim	4:01	47
Path	Apocalyptica	3:06	37
Im Herz	Kubrick feat. Xavier Naidoo	3:59	49
Silence	DJ Tomcraft	4:19	45
Firstclass Suicide	Anna Luca	4:23	48
Cloudburst—Grand Canyon Suite	Ferde Grofé	7:42	47
My Heart Is Empty	Garda	3:34	49
Mother and Child	Sara Lugo	4:01	50
Hiroshima	Greta	3:49	43

*Frequency denotes the number of occurrences of each song over all participants. Numbers differ because songs were presented in random order and the first song for each participant was used as a training stimulus and not included in the analyses. Moreover, songs that were known to a participant prior to the study were also excluded*.

### Apparatus

The laboratory room was equipped with six tables, each with a comfortable chair, a computer with 17-in monitor, optical mouse, and Sennheiser HL 270 stereo headphones. Up to six participants at a time could be seated. They were separated from each other by wooden screens. Lights were dimmed. An “emoslide” java program, which was designed for the study, played the songs in random order and simultaneously measured the moment-to-moment ratings of subjectively experienced intensity of felt emotions. Participants had to move a digital slider with the mouse to continuously rate their experienced emotional intensity on a scale labeled with *no emotion at all* at the bottom and *very intense emotion* at the top. The length of the scale on the monitor was 105 mm. Participants' ratings were recorded with a sampling rate of 10 data points/s (10 Hz; see Nagel et al., 2007). Data readout ranged from 0 (*no emotion at all*) to 100 (*very intense emotion*) in 101 possible steps. Retrospective evaluation of overall emotional intensity was measured by participants' single global rating for each song with a pencil on the same scale printed on paper. Furthermore, for every song, participants had to indicate if they had ever listened to it before.

### Procedure

When participants had been seated in front of the computers, they received instructions regarding the purpose of the study, the use of headphones, volume settings, the digital slider, and the questionnaires. Furthermore, participants learned from information given on the computer screen that the study was about the individual course of their experienced emotions. They should continuously observe the intensity of emotions they felt and indicate their ratings on the scale on the screen by moving the digital slider, which they were able to become familiar with before the first experimental song started. After participants started the session, the 11 songs were played in random order and moment-to-moment ratings of emotional intensity were recorded. After each song, there was a short break of 10 s, automatically followed by the next song.

When they had listened to all the songs, participants were told that the second part of the study was about the overall intensity of emotions felt for each song. As some time had passed since they had listened to each specific song, they were told that short representative excerpts of about 20 s of each song (snippets) should help them recollect the music. They were instructed to remember and retrospectively evaluate the intensity of emotions elicited by the respective song in general. After participants started the session, the 11 snippets were played in the same individual order as the songs were played earlier. After each snippet, participants had time to give one overall evaluation of emotional intensity per song on a paper version of the scale. They also were to indicate whether they had heard any of the songs prior to the study. The procedure led to a certain time delay between listening with moment-to-moment rating and making the retrospective evaluation, which was desired to reduce recency effects due to participants still having their rating profiles in mind. Finally, participants completed a questionnaire on their personal data and musical habits and were then debriefed.

### Statistical analyses

**Figure 1:**
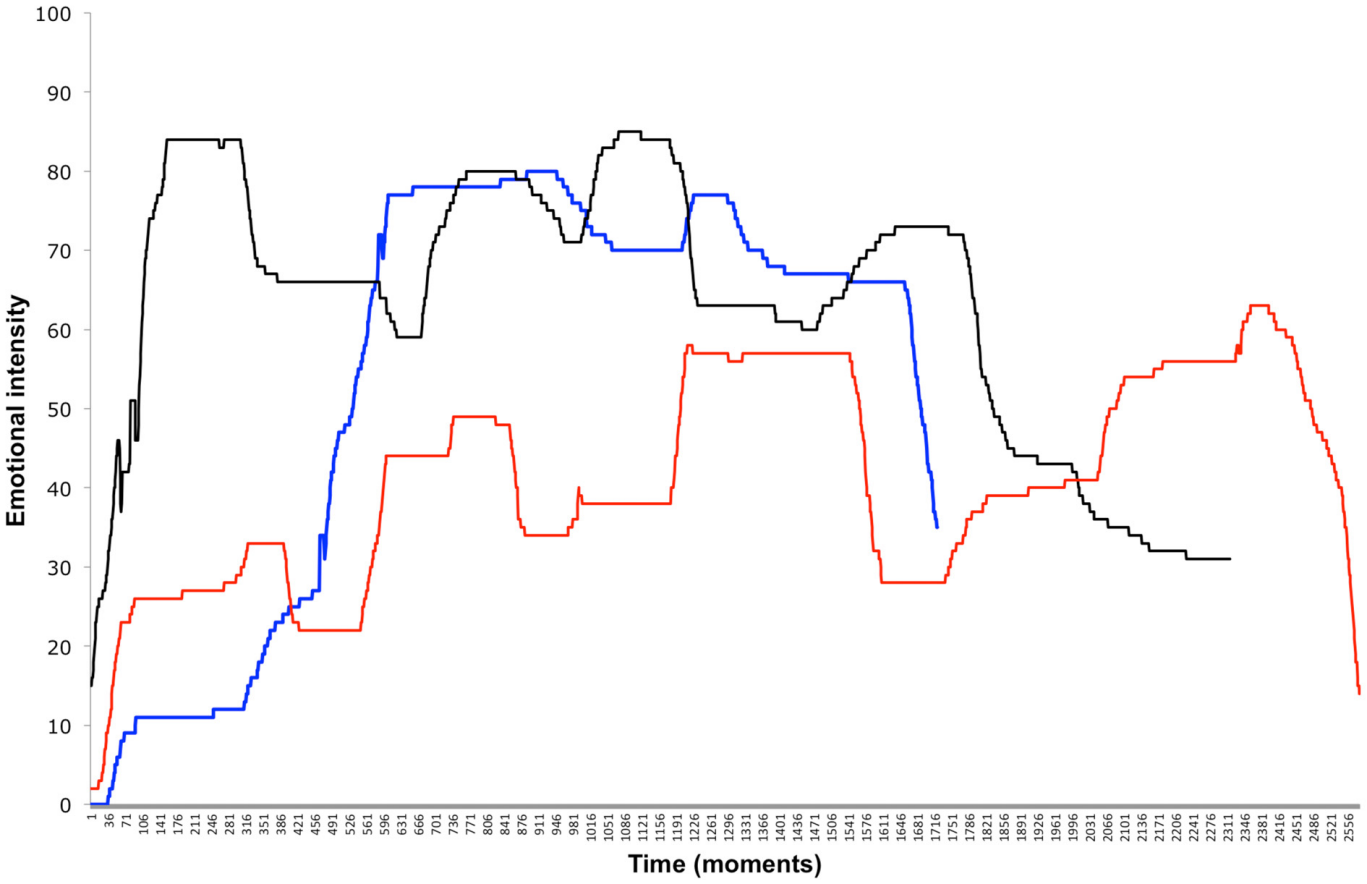
**The temporal profiles of emotional intensity ratings for three exemplary songs from one exemplary participant**.

Every participant generated continuous moment-to-moment emotional intensity data and a corresponding retrospective rating of overall emotional intensity for each of the 11 songs. Only songs that participants had never listened to before were included in the analysis. The first song every person listened to was excluded because it was used for training the continuous self-reporting by using the slider. As songs were presented in random order, the exclusion affected each of the 11 songs with about the same frequency (the resulting absolute frequency with which each song was included in the analyses is shown in Table 1). Figure 1 gives an example of the temporal profiles of three exemplary songs from one exemplary participant. As can be seen, a profile can exhibit more than only one peak. Note that lines are of different lengths because the songs differed in their lengths and thus in the number of “moments.”

**Figure 2:**
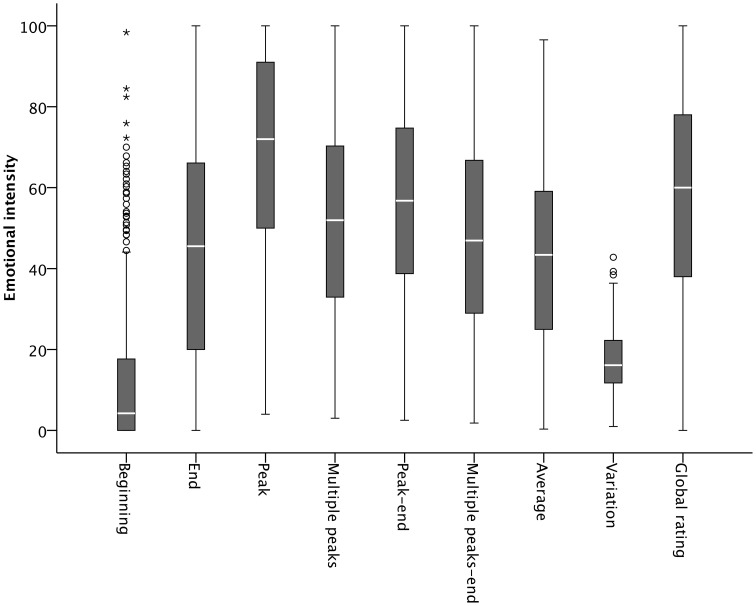
**Boxplots showing the distributions of the moment-to-moment parameters and the global rating of emotional intensity, calculated across all participants and all songs**. Note that *sum* and *number of multiple peaks* are not included because they have a different scale.

Beginning, peak, multiple peaks, end, peak–end, multiple peaks–end, number of multiple peaks, sum, average, and variation variables were calculated for every song for every person. *Beginning* of a song was defined as the period from 5 to 15 s after the onset of a song. Unfolding of emotions over time and response latencies for corresponding ratings are thought to take place within about 5 s of stimulus onset (Sloboda and Lehmann, 2001; Nagel et al., 2007). In addition, the slider on the screen was positioned at the bottom of the scale when each trial started. If the participants could not be expected to move the slider within the first seconds, this would produce zero values for the beginning that should not be interpreted as an absence of emotional intensity during this period, however. Therefore, to obtain a reliable measure of a song's onset of emotional intensity, *beginning* was calculated by the average of those 10 s. *End* of a song was defined as the last 10 s and was calculated by the average of ratings of the last 10 s. *Peak* of a song was defined as the maximum value of the whole temporal profile. The *multiple peaks* variable was defined as the average of all moment ratings that represented a local maximum^2^. *Peak–end* of a song was calculated by the average of the peak and end values. As an alternative peak–end measure, a *multiple peaks–end* variable was calculated by the average of the multiple peaks and end values. *Number of multiple peaks* was the number of the local maxima during the whole piece. *Sum* was defined as the sum of the intensities of all the single moments of the experience. *Average* of a song was defined as the arithmetic mean of all single values. *Variation* of a song was defined as the standard deviation of the temporal profile. The boxplots in Figure 2 show the descriptive statistics of all the parameters and the overall evaluation (*global rating*). Note that *sum* (*M* = 104.659; *SD* = 60.842) and *number of multiple peaks* (*M* = 7.6; *SD* = 3.4) are not included in Figure 2 because they had a different scale.

To analyze which of these parameters contribute most to the explanation of variance of the overall evaluation, we first calculated Pearson correlation coefficients. Our prediction was that *average* would exhibit the highest correlation to the overall evaluation. In addition, we ran a multiple regression analysis, in order to compare the relative impact of the most important parameters. Since many of the parameters we calculated can be expected to be affected by multicollinearity (e.g., *sum* will be highly correlated with *average*), we were not able to include all of the parameters in a simultaneous regression analysis. However, at least the two most important parameters should be taken into account in any case: *average*—as a measure of the temporal integration theory—and *peak–end*—as a potential measure of the duration neglect theory. Our prediction was that *average* is a better predictor of the overall evaluation than *peak–end*. Note that the multiple regression analysis is based on variances that might originate from different levels, that is, different participants and different songs. Data that are organized in such a hierarchical structure might produce level effects, leading to artificial correlations that are due to different response patterns (e.g., participants with a restrained vs. participants with a permissive response behavior). Therefore, to control for the different variance components, we ran a multiple regression analysis using hierarchical linear modeling (with the software HLM 7; Raudenbush et al., 2004).

## Results

### Correlation analysis

**Figure 3:**
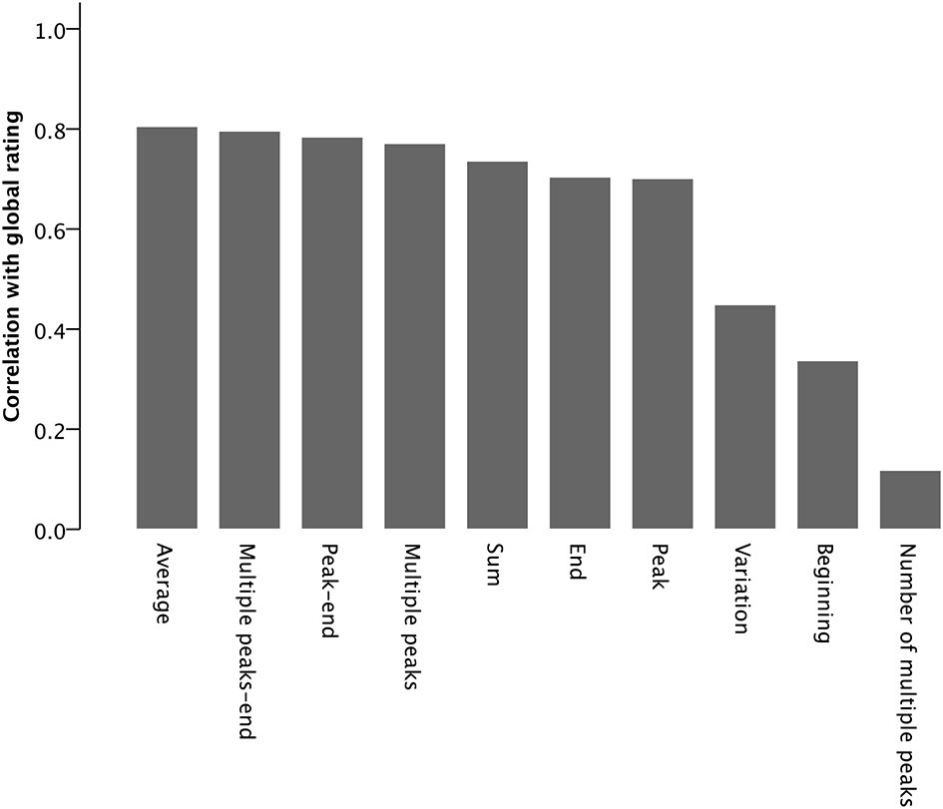
**Pearson correlations between the overall evaluation and all the parameters of moment-to-moment experience (*N* = 507)**.

Table 2 and Figure 3 show the results of the correlation analysis. While *average* is most highly correlated with the global rating—which corresponds to our prediction—most of the remaining parameters also exhibit high correlations: *end, peak, multiple peaks, peak–end*, *multiple peaks–end* and *sum*. *Beginning*, *number of multiple peaks*, and *variation* were only moderately correlated with the global rating. That is, these results do not clearly speak for or against one specific theory regarding the emergence of the overall evaluation. Considering these results, one might suppose that listeners build a moving average while listening to the music but that the average is affected by moments of specific importance—peak or multiple peaks, respectively, and end—that can be combined to a peak–end measure. Since we were interested in a direct comparison of the contributions of parameters to explaining the variance of the global rating we additionally ran multiple regression analyses.

**Table 2:** **Pearson correlations between the overall evaluation and all the parameters of moment-to-moment experience (*N* = 507)**.

**Parameter**	**Beginning**	**End**	**Peak**	**Multiple peaks**	**Peak–end**	**Multiple peaks–end**	**Number of multiple peaks**	**Average**	**Sum**	**Variation**
*Global rating*	*0.337*	*0.704*	*0.701*	*0.771*	*0.784*	*0.796*	*0.118*	*0.805*	*0.736*	*0.449*
Beginning		0.276	0.362	0.440	0.354	0.382	0.297	0.478	0.442	−0.122
End			0.604	0.698	0.905	0.935	0.096	0.763	0.700	0.634
Peak				0.903	0.885	0.805	0.241	0.849	0.785	0.755
Multiple peaks					0.890	0.908	0.179	0.963	0.880	0.579
Peak–end						0.974	0.184	0.897	0.827	0.605
Multiple peaks–end							0.146	0.927	0.849	0.497
Number of multiple peaks								0.197	0.355	0.032
Average									0.926	0.483
Sum										0.421

*All coefficients are significant at p < 0.001, except for global rating and number of multiple peaks (p = 0.008), beginning and variation (p = 0.006), end and number of multiple peaks (p = 0.031), number of multiple peaks and variation (p = 0.474)*.

In addition, we also calculated the correlation between remembered overall intensity (*global rating*) and *liking*, yielding a strong positive correlation of *r* = 0.63 (*p* < 0.001).

### Multiple regression analysis

Due to multicollinearity, we could not include all of the parameters in a single simultaneous regression analysis. For instance, the variable *peak–end* is a nearly perfect linear combination of the variables *peak* and *end*, so these three variables cannot be included in a regression analysis simultaneously. Thus, we had to decide which of the parameters to include. Given the theoretical arguments raised in the Introduction, we were obliged to include at least *average* and a *peak–end* measure. Regarding the peak–end measure, it was difficult to decide whether to use the average of *peak* and *end* values or the average of *multiple peaks* and *end* values, because both measures were highly correlated with the global rating. We therefore calculated two separate models, one including *peak–end* and another one including *multiple peaks–end*. From the remaining parameters, there were only two left that were not affected by multicollinearity: the number of multiple peaks and the variation of the song's profiles. So we eventually included *average*, *peak–end* or *multiple peaks–end*, respectively, *number of multiple peaks*, and *variation* in simultaneous regression analyses.

To check for potential level effects, we first calculated the intraclass correlation, obtaining a value of *r* = 0.11, which indicated the use of hierarchical linear modeling. Each of the two regression models accounts for about 70% of the variance of the overall evaluation. The specific influences of the four predictors in each model are shown in Table 3. In both models, *average* and *peak–end* or *multiple peaks–end*, respectively, turned out to be significant predictors, with *average* yielding much larger regression coefficients than *peak–end* or *multiple peaks–end*, respectively. *Variation* and *number of multiple peaks* did not turn out to be significant predictors of the global rating.

**Table 3:** **Regression coefficients of a selection of distinct parameters of moment-to-moment experience for their influence on the overall evaluation (*N* = 507)**.

**Parameter**	**β**	***p***	***R*^2^**
*Model 1*			0.69
Average	0.59	<0.001	
Peak–end	0.26	<0.001	
Variation	0.02	0.56	
Number of multiple peaks	0.01	0.61	
*Model 2*			0.70
Average	0.58	<0.001	
Multiple peaks–end	0.25	0.002	
Variation	0.06	0.08	
Number of multiple peaks	0.02	0.37	

## Discussion

The aim of the present study was to answer the question of how people remember and evaluate the emotional intensity of past musical experiences. To investigate which parameters of past musical experiences influence retrospective overall evaluation of emotional intensity most, several parameters of musical experience's temporal profile of emotional intensities were taken into account: beginning, peak, multiple peaks, end, peak–end, multiple peaks–end, number of multiple peaks, sum, average, and variation. A correlation analysis showed that all of these parameters are significantly correlated with the overall evaluation, with *peak*, *multiple peaks*, *end*, *peak–end*, *multiple peaks–end*, *sum*, and *average* exhibiting large correlation coefficients. *Average* was the parameter most highly correlated with the global rating, but that correlation was only slightly larger than those between the global rating and other parameters such as *peak–end* or *multiple peaks–end*. When a selection of non-multicollinear parameters was taken into account as simultaneous predictors of the global rating, *average* emerged as the most influential variable. A second significant predictor—with a much smaller regression coefficient—was the peak–end variable. For the peak–end variable, it did not matter whether it was calculated from either the average of one *peak* (the highest value of the temporal profile) and the end value or *multiple peaks* and the end value. This indifference is also highlighted by the non-significant influence of the number of multiple peaks on the global rating. Thus, for the overall impression about the emotional intensity of a musical experience, it seems to be essential that there is an outstanding peak but it appears to make no difference if there is only one peak or a series of multiple peaks.

Connecting these results to the theoretical approaches discussed in the introduction provides us with an interesting picture. As we have pointed out, there are two main competing theories: one that proposes that every single moment of an experience is integrated when an overall evaluation is processed (temporal integration) and one that proposes that this is the case for only some specific single moments (duration neglect). Our results demonstrate that the average of all experienced moments is the best predictor for the overall evaluation. In addition, however, some specific moments of the experience appear to have an additional influence on the final evaluation. We can thus conclude that some specific moments (such us peaks or the end) or an average of such moments (such as peak–end or multiple peaks–end) do play a role in building an overall evaluation but that they are neither sufficient for this evaluation nor the most important elements of an experience. As we argued earlier, listeners might continuously update the level of emotional intensity over the course of a listening experience. Longer passages of weak emotional intensity will lower the moving average and thus the retrospective evaluation, while longer passages of high emotional intensity will elevate the moving average and the subsequent evaluation. However, it seems reasonable that moments of outstandingly high intensity (such as the peak or multiple peaks) and the moments at the very end (which “benefit” from a recency effect) have an additional impact on the overall evaluation. There may be two stages where this can occur. One possibility is that moments of outstanding intensity affect the continuous calculation of the moving average with a higher weight than any other moments *online*, that is, *during the course of the listening experience*. There is a finding by Rozin et al. (2004) that speaks for this conjecture. These authors found that steeper slopes in the profile of the moment-to-moment emotional intensity led to higher overall evaluations. This might indicate that steeper slopes presage a peak that subsequently affects the processing of an average more intensely. An alternative possibility is that listeners continuously calculate an average, which is adjusted by peak and end moments only *afterward*, that is, *when the listening experience is over*. In this case, listeners would have to keep the whole temporal profile in mind because they cannot identify peaks until the experience has come to an end. So they would calculate the average of *peak–end* or *multiple peaks–end* only in retrospect and subsequently use this value to adjust the initial average. Cojuharenco and Ryvkin (2008) have also argued that moment-to-moment bits of information of an experience do not get lost—people use them very well for processing an average value—but that peaks[s] and end are nevertheless important information people might pay particular attention to when building their subsequent evaluation. With the present data we are not able to adjudicate between these alternative hypotheses. One can question, however, how likely it is that listeners will hold a correct and unbiased representation of the whole temporal profile in mind. It is more likely that what listeners remember is an evaluation that was left at the end of a listening experience and achieved through a weighted averaging of the experience.

The slope effect found by Rozin et al. (2004) had also led us to incorporate a measure of variation in the prediction of the overall evaluation. Although exhibiting a medium correlation with the global rating, this parameter did not specifically contribute to the prediction of the global rating. Thus, bringing more variation into an experience does not seem to lead to a higher evaluation of the experience in retrospect.

If our conclusions are correct they should affect our understanding of retrospective affective judgments in general, not only for musical stimuli. It seems reasonable to suggest that most kinds of experiences are remembered and evaluated by a combination of average and a peak–end variable. Moreover, this might account for the heterogeneous findings in past research (see above) and it seems worthwhile to replicate studies with painful or pleasurable experiences while taking into account all the potential parameters that might influence an overall evaluation.

The memory and evaluation of affective intensity is clearly of interest to performers and composers, whether they want to arrange a single piece of music that leaves an intense overall memory, affectively powerful musical experiences consisting of several movements, or a number of pieces into an album. Taking the results of the present study into account, when composing a piece of music that is intended to be remembered as emotionally intense one should arrange for an overall high level of emotional intensity or at least for a high peak or several high peaks, respectively, and an emotionally intense end. People's attitudes and behavior (how much they like a song, whether they want to attend a concert or buy a CD) are likely to be influenced by the overall emotional intensity of a musical experience they have kept in mind. We have found a strong positive correlation between remembered emotional intensity and liking. Studying the time course of emotional intensity during concerts or when listening to a whole set of pieces remains an interesting task for future research, at any rate (see also Ariely and Zauberman, 2000).

Note that we defined the *peak* as the most emotionally intense moment and *multiple peaks* as the average of all local maxima of moment-to-moment intensity of a musical experience. We believe, however, that the peak concept needs some theoretical clarification in the future, at least with regard to music. Specifically, it remains to be clarified to what degree peak experiences are comparable to frisson or chill experiences (e.g., Goldstein, 1980; Sloboda, 1991; Panksepp, 1995). There are some candidate musical characteristics that can elicit chills (such as crescendos, the beginning of a new part, onset of a voice; see Huron, 2006; Grewe et al., 2007a), which might play an important role in the memory of past musical experiences.

Not least, we would argue that attitudes and behavior regarding music depend not only on emotional intensity but also—and particularly—on pleasantness. Hence, a study that investigates the time course of pleasantness and the influence of its parameters on the global evaluation of pleasantness would tie in well with the present work. Unlike the results we found in the present study, results of an investigation of pleasantness could reveal a much stronger impact of the beginning of a musical experience, since we all know that whether we like a piece of music or not is decided quite quickly, after just a few seconds (see e.g., Zajonc, 1980; Salimpoor et al., 2013).

### Conflict of interest statement

The authors declare that the research was conducted in the absence of any commercial or financial relationships that could be construed as a potential conflict of interest.

## Footnotes

^1^We should mention that a special form of the temporal integration model claims that the duration of the experience itself matters. That is, any affect, be it strong or weak, always simply increases the “proportion” of affect felt—as long as the affect does not change its valence. This leads to the conclusion that the longer an experience lasts, the greater the remembered pleasure or displeasure should be, which has been referred to as temporal monotonicity. However, this conjecture does not appear to be very plausible and it has received no empirical support.

^2^A value was defined a local maximum when there were no higher values within a 20 s window around that value AND that value lay above the moving average, which was also calculated using a 20 s window. Twenty seconds were chosen as a reasonable range because peaks usually last between 2 and 20 s (see Grewe et al., 2007b).
